# Experimental identification and in silico prediction of bacterivory in green algae

**DOI:** 10.1038/s41396-021-00899-w

**Published:** 2021-03-02

**Authors:** Nicholas A. Bock, Sophie Charvet, John Burns, Yangtsho Gyaltshen, Andrey Rozenberg, Solange Duhamel, Eunsoo Kim

**Affiliations:** 1 Lamont-Doherty Earth Observatory, Columbia University, Palisades, NY USA; 2 Division of Invertebrate Zoology and Sackler Institute for Comparative Genomics, American Museum of Natural History, New York, NY USA; 3 Bigelow Laboratory for Ocean Sciences, East Boothbay, ME USA; 4 Faculty of Biology, Technion - Israel Institute of Technology, Haifa, Israel; 5 Now at Department of Molecular and Cellular Biology, The University of Arizona, Tucson, AZ USA

**Keywords:** Microbial ecology, Cellular microbiology

## Abstract

While algal phago-mixotrophs play a major role in aquatic microbial food webs, their diversity remains poorly understood. Recent studies have indicated several species of prasinophytes, early diverging green algae, to be able to consume bacteria for nutrition. To further explore the occurrence of phago-mixotrophy in green algae, we conducted feeding experiments with live fluorescently labeled bacteria stained with CellTracker Green CMFDA, heat-killed bacteria stained with 5-(4,6-dichlorotriazin-2-yl) aminofluorescein (DTAF), and magnetic beads. Feeding was detected via microscopy and/or flow cytometry in five strains of prasinophytes when provided with live bacteria: *Pterosperma cristatum* NIES626, *Pyramimonas parkeae* CCMP726, *Pyramimonas parkeae* NIES254, *Nephroselmis pyriformis* RCC618, and *Dolichomastix tenuilepis* CCMP3274. No feeding was detected when heat-killed bacteria or magnetic beads were provided, suggesting a strong preference for live prey in the strains tested. In parallel to experimental assays, green algal bacterivory was investigated using a gene-based prediction model. The predictions agreed with the experimental results and suggested bacterivory potential in additional green algae. Our observations underline the likelihood of widespread occurrence of phago-mixotrophy among green algae, while additionally highlighting potential biases introduced when using prey proxy to evaluate bacterial ingestion by algal cells.

## Introduction

Mixotrophic (or phago-mixotrophic) phytoplankton are microorganisms capable of using both autotrophy (i.e., photosynthesis) and phagotrophy (e.g., bacterivory) to obtain the carbon and nutrients required to support cellular processes. Because of their ability to utilize different trophic modes, mixotrophs are unique and important links in marine microbial food webs. The degree to which they utilize autotrophy vs. phagotrophy can have wide-ranging ecological consequences, potentially influencing microbial trophic structure and community stability [1, 2] in addition to community primary production and carbon cycling [3].

The occurrence of mixotrophy has been long recognized in dinoflagellates [4], haptophytes [5, 6], cryptophytes [7, 8], and chrysophyceans [9–12]. In addition, there is a growing sense for potential major roles played by green algae in bacterivory in oceanic and other aquatic environments. Bell and Laybourn-Parry [13], for example, reported ingestion of both fluorescently labeled microspheres and DTAF-labeled bacteria in Arctic strains of *Pyramimonas*. Anderson et al. [6] reported ingestion of DTAF-labeled bacteria by cells in two *Nephroselmis* strains, while McKie-Krisberg et al. [14, 15] reported ingestion of fluorescent microspheres by both pico- and nano-sized green algae. Paasch [16] additionally reported stimulated growth in cultures of *Cymbomonas tetramitiformis* inoculated with co-cultured bacteria compared to controls.

Accounting for contributions to total bacterivory made by green algae is crucial to understanding aquatic microbial food web structure and functioning. In particular, prasinophytes—a paraphyletic assemblage of green algae often characterized by the presence of organic scale covering on the cell body and flagella—are globally distributed and in some cases may account for the majority of phototrophic cells in picophytoplankton size classes [17–19] across biogeochemically distinct oceanic regions. Moreover, because the ancestors of Chloroplastida (or Viridiplantae)—comprising green algae and land plants—were presumably phagotrophic, the structure, physiology, and genes that characterize green algal bacterivory may also help illuminate the origin and early history of this highly successful group [20]. The discovery that *Cymbomonas* has a complex feeding structure that includes the duct system (originally suggested to be involved in the transportation of scales to the cell surface) and an acidic spherical compartment where digestion takes place, points to the possibility that other prasinophytes presenting a similar structural organization might also be capable of ingesting bacteria [21].

The objective of this study was to explore bacterivory in early diverging green algae by using a combination of experimental assays and gene-based trophic prediction models. Five prasinophyte strains were selected for feeding assays based on morphology, phylogeny, and/or preliminary in silico prediction results using an earlier version of the trophic model presented in Burns et al. [22]. The selected strains include three pyramimonadophycean strains (*Pterosperma cristatum* NIES626, *Pyramimonas parkeae* CCMP726, and *Pyramimonas parkeae* NIES254); a nephroselmidophycean strain (*Nephroselmis pyriformis* RCC618); and a mamiellophycean strain (*Dolichomastix tenuilepis* CCMP3274). The occurrence of bacterivory in these strains was tested via the detection of ingested fluorescently labeled bacteria (FLB) via epifluorescence microscopy and flow cytometry. The combined use of these techniques allowed for both the visual confirmation of ingestion via microscopy in addition to the rapid quantification of changes in cellular fluorescence via cytometry [23]. Further, we explored the potential for phagotrophy in diverse green algae using the gene-based trophic model from Burns et al. [22], utilizing a wealth of existing transcriptome and genome data.

## Materials and methods

### Selection of algal strains

All genera of green algae selected for this study are marine flagellates with scales covering the plasma membrane of the cell body and flagella [24, 25]. Of these, *Pterosperma* and *Pyramimonas* are closely related to *Cymbomonas*, all belonging to the Pyramimonadophyceae; members of which display a duct system or its equivalent that is associated with complex cytoskeletal elements [21, 24, 26]. In addition, *Pterosperma* was previously shown to endocytose labeled glucans and be positive for acid phosphatase, a conserved component in degradative cellular compartments [27]. *P. parkeae* CCMP726 and *D. tenuilepis* CCMP3274 were predicted to be mixotrophs using an earlier version of gene-based models as presented in Burns et al. [22]. Finally, *N. pyriformis* RCC618 represented a third prasinophyte family, from which other strains (K-0557, K-0556) have been shown to be bacterivorous [6].

### Growth of algal strains

Algal cultures were maintained at 17 °C on a 12-h light/dark cycle. Growth irradiance was ~80 µE m^−2^ s^−1^ for all cultures. All algal cultures were monoprotistan and grown in the presence of undefined bacterial flora. For cytometry experiments, aliquots of maintenance cultures in mid-exponential growth were transferred in a 1:10 final dilution to 20 mL of either f/2 (hereafter nutrient replete) or f/20 (hereafter nutrient limited) growth medium. Nutrient-replete media was prepared as described in Guillard [28]. Nutrient-limited media was prepared by diluting nutrient-replete media 1:10 in artificial seawater (ASW). As green algae do not generally require silica, the silica was not added to the growth media. To determine the growth phase of the cultures, in vivo fluorescence measurements were taken at regular intervals using a Turner AquaFluor fluorometer (Turner Designs, San Diego CA). Feeding experiments were conducted when the nutrient-replete treatments were in mid to late exponential growth phase.

### Preparation of fluorescently labeled bacteria

FLB were prepared from cultures of *Pelagibaca bermudensis* HTCC2601 [29, 30], a widely distributed marine bacterium chosen for its small size (1.2–2.3 µm long) relative to the algal strains used. Bacterial cultures were maintained at 27 °C in ASW enriched with glucose (0.2% w/v) and yeast extract (0.5% w/v). Bacterial cultures were generally in late exponential or stationary growth phase at the time of labeling. To obtain appropriate cell densities for labeling, growth medium was removed from culture flasks using a serological pipette, and the remaining bacterial pellet was resuspended in 1 mL of ASW. *P. bermudensis* cells are non-motile [29] and precipitated naturally during culture growth, eliminating the need for centrifugation to obtain a cell pellet. Triplicate 1 mL subsamples of the cell suspension were labeled with 5 µL SYBR Green I DNA dye (Thermo Fisher Scientific, Waltham MA; Cat. # S785) diluted to a final concentration of 1:10,000, and enumerated via flow cytometry. For the preparation of live labeled FLB the initial cell suspension was diluted in ASW to a final cell abundance of 1 × 10^9^ cells mL^−1^ and a final volume of 1 mL. The remainder of the initial suspension was then used for the preparation of heat-killed FLB.

Live FLB were prepared by labeling bacterial cells with CellTracker Green CMFDA (Thermo Fisher Scientific, Waltham, MA; Cat. # C7025): a low toxicity cytoplasmic stain [31]. Working solutions of CellTracker Green CMFDA (hereafter CT) were prepared in accordance with manufacturer guidelines and added to one of the dilute cell suspensions to a final concentration of 10 µM. The CT-labeled FLB suspension (hereafter CT-FLB) was then vortexed for 1–2 s and incubated for 3 h in a water bath at 37 °C. Viability tests conducted with *P. bermudensis* indicated bacterial cells were viable after incubation at 37 °C (See Supplementary Materials SM1.1 and SM1.2; Supplementary Fig. 1). Heat-killed FLB were labeled with 5-(4,6-dichlorotriazin-2-yl) aminofluorescein (5-DTAF, Thermo Fisher Scientific, Waltham, MA; Cat. # D16) and prepared as per Sherr et al. [32] and Vazquez-Dominguez et al. [33] (hereafter DTAF-FLB). The DTAF-FLB were then vortexed and incubated for 2 h in a water bath at 60 °C. *P. bermudensis* viability tests indicated bacterial cells to be no longer viable following incubation at temperatures above 45 °C (See Supplementary Materials SM1.1 and SM1.2; Supplementary Fig. 1).

To remove excess dye following incubation, each cell suspension was filtered through a 25-mm diameter 0.2-μm porosity polycarbonate filter and rinsed 3× with 1 mL aliquots of ASW. Each filter was transferred to a microcentrifuge tube with 1 mL ASW and vortexed for 30 s to detach cells from the filter. The resulting cell suspensions were then transferred to a sterile microcentrifuge tube and stored at 4 °C until the start of experiments. When experiments were conducted more than 12 h after the preparation of FLB, wash steps were repeated immediately before the start of experiments. All experiments were conducted within 24 h of the preparation of FLB.

### Flow cytometric examination of bacterivory by green algae

All cytometric assays of bacterivory were conducted using a Guava Easycyte flow cytometer (MilliporeSigma, Burlington, MA). For each algal strain tested, triplicate subsamples were prepared from both nutrient-replete and nutrient-limited culture flasks for two experimental treatments: one inoculated with CT-FLB and one inoculated with DTAF-FLB. To ensure algal cell density did not exceed the maximum number of countable cells by the Guava Easycyte flow cytometer, initial algal cell densities in experimental cultures were enumerated, with subsamples then being prepared in ASW to a final volume of 500 µL and final algal density of less than 2 × 10^5^ cells mL^–1^. Treatments were then inoculated with either CT-FLB or DTAF-FLB to final FLB concentrations at 10–50% of those for unlabeled bacteria. As a negative control (hereafter CT-FLB + PFA), an additional set of triplicate subsamples from both nutrient-replete and nutrient-limited culture flasks were fixed with 32% electron microscopy grade paraformaldehyde (PFA) to a final concentration of 4% 1 h prior to inoculation with CT-FLB. Only one replicate was prepared for *P. parkeae* NIES254 and CCMP726 treatments inoculated with DTAF-FLB. All treatments were kept in a bench-top incubator, at the same temperature and light intensity as used in the algal growth chamber, for the duration of experiments.

**Fig. 1:**
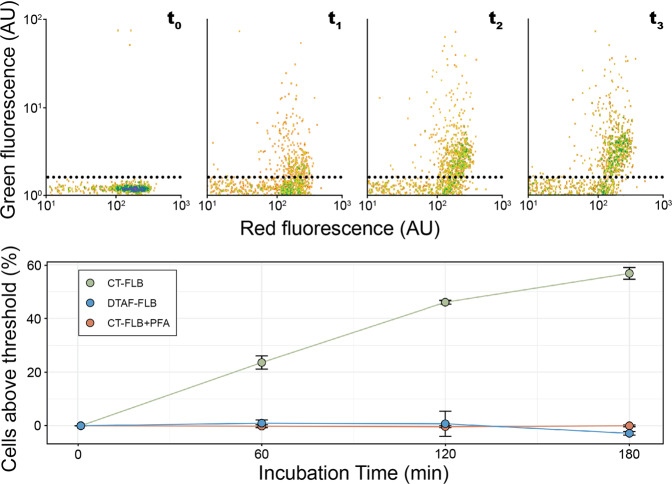
Example of cytometry results based on experiment conducted with *Pterosperma cristatum*. Top panel: cytograms corresponding to time points t_0_–t_3_ in treatment inoculated with CT-FLB. Log-transformed red fluorescence plotted on the *x-*axis in arbitrary units (AU); log-transformed green fluorescence plotted on the *y*-axis in arbitrary units. For clarity, all cytograms were scaled to fill the plotting area and centered on the *x-*axis. As such, origin of *x-*axis is 10^1^. Dashed line indicates the position of GF_prior_. Bottom panel: change in per_fed_ over 180 min incubation. Colors correspond to prey type used. Green: CT-FLB; blue: DTAF-FLB; red: CT-FLB in sample fixed with PFA (CT-FLB + PFA). Error bars correspond to standard error (*n* = 3).

Algal cell populations were identified and enumerated based on chlorophyll red fluorescence and forward scatter signals. Uptake of FLB by algal cells was inferred from changes in the green fluorescence signal of algal cell populations over 3 h (Fig. 1; Supplementary Fig. 2). Green fluorescence was measured cytometrically for each subsample prior to inoculation with FLB to determine the maximum baseline green fluorescence of algal cultures (GF_prior_; Supplementary Fig. 2). The duration of experiments was selected based on low ingestion rates in several of the strains used, making it not typically possible to resolve feeding within intervals less than 1 h. Subsamples were then analyzed immediately following inoculation with FLB (t_0_) and then at 1-h intervals for 3 h (t_1_, t_2,_ t_3_). An increase in background fluorescence was observed immediately after addition of FLB for all algal cultures and accounted for by measurements at t_0_ and then subtracting these values from subsequent times points. For each sample analyzed by flow cytometry, the total algal cell abundance (cells_total_) was recorded. The number of cells with green fluorescence exceeding GF_prior_, indicating ingestion of labeled prey, was recorded as cells_fed_. The percentage of cells_total_ having ingested FLB (per_fed_) was calculated as (cells_fed_/cells_total_) × 100. The difference between per_fed_ at t_0_ and per_fed_ at t_3_ was calculated and expressed as per_Δ_. Green fluorescence of FLB was measured at t_0_ and t_3_ in CT, DTAF, and CT-FLB + PFA treatments to confirm the stability of FLB fluorescence following fixation (see Supplementary Fig. 3 for representative cytograms).

### Epifluorescence microscopic examination of bacterivory by green algae

Algal cultures were incubated in 6 × 8 multiwell plates (CellTreat Scientific Products, Pepperell, MA) for 15–90 min in the algal growth chamber with concentrations of CT-FLB or DTAF-FLB similar to those in the cytometry experiments described above. The prasinophyte *Cymbomonas tetramitiformis* PLY262 and haptophyte *Diacronema lutheri* RCC180, known phago-mixotrophs, were used as general positive controls for FLB ingestion. For strain-specific negative controls to illustrate absence of feeding, the algae were killed in 4% PFA prior to adding CT-FLB. For strain-specific negative controls to check for possible false staining with residual CT stain, the algae were inoculated with the 0.2 µm-filtered supernatant from the last CT-FLB washing step. After incubation, the live algal cells were observed under an epifluorescence microscope (Axiovert 100 M, Carl Zeiss, Germany) equipped with filter sets for FITC detection (excitation 470/40, emission 535/40) and chlorophyll detection (excitation 480/30, emission 600LP) to reveal the CellTracker Green or DTAF and natural chlorophyll autofluorescence, respectively. Micrographs were captured with a DP73 camera (Olympus, Japan). The presence of bright green fluorescence inside the algal cell was interpreted as a positive feeding signal. All five prasinophyte strains are characterized by rapid swimming behavior, which, combined with their small cell size, impeded precise counts of ingested bacteria. Furthermore, when exposed to chemical fixatives, including glutaraldehyde, formaldehyde, a mixture of the former two, Lugol’s iodine solution and Misky’s fixative, the cells would rapidly (within 2 s) lose fluorescence and/or show signs of egesting the fluorescent contents of their vacuoles. Therefore, all observations were carried out on live cells and consisted in the qualitative assessment of feeding behavior.

### Predictions of phago-mixotrophy using a gene-based model

Predicted protein data from 19 genome and 71 transcriptome assemblies of green algal species, selected as shown below, were tested for phagocytotic potential using a gene-based model formulated by Burns et al. [22]. The majority of the green algal gene sets used in this study originated from the latest version of transcriptome assemblies generated as part of the Marine Microbial Eukaryote Transcriptome Sequencing Project (MMETSP; doi:10.5281/zenodo.3247846) [34, 35] and the Plant 1000 Transcriptome Project (1KP; http://www.onekp.com/) [35–37]. Coding sequences for all of the transcriptomes were re-annotated using TransDecoder v. 5.5.0 (https://github.com/TransDecoder/TransDecoder). For genome assemblies, whenever gene annotations were not available from the source databases, de novo gene prediction was performed with GeneMark-ES v. 4.38 [38] in self-training mode or using models trained on closely related species in case of highly fragmented assemblies. To increase the probability of detecting gene products, protein sets were merged on the level of strains. Completeness of protein sets was assessed with BUSCO v. 4.0.5 [39] against the full eukaryote (odb10) lineage. Only the sets with less than 32% missing BUSCO genes were kept for the prediction model, except for the *Cymbomonas* sp. M3265 and *Mesostigma viride* SAG50-1 transcriptomes, which were kept for the analysis despite missing 33.0 and 41.5% of eukaryote orthologs, respectively. We also excluded the *Aphanochaete repens* M2226 protein set showing contaminant sequences matching to a lobose amoeba.

Computational prediction of phagocytotic, photosynthetic, and prototrophic capabilities used the predictTrophicMode tool described in detail in Burns et al. [22]. The code is available at https://github.com/burnsajohn/predictTrophicMode and was run in the default mode after scoring all test genomes and transcriptomes against the hidden Markov models (HMMs) that form the core of the predictTrophicMode tool. Due to broad sampling of eukaryote diversity for the training genomes, the tool is capable of detecting phagocytotic signatures in divergent and novel lineages, such as *Mantamonas* [22] and the newly discovered *Rhodelphis* [40]. The predictor is a classifier that was trained by first grouping, by unsupervised clustering, all proteins in 35 complete eukaryote genomes using all vs. all blast followed by MCL clustering. Clusters that satisfied a diversity criterion of containing genes from at least 3 different genomes out of 35 were aligned and converted to HMMs, establishing 14,095 gene models. Individual genes enriched in groups of organisms that share a trophic mode were inferred by grouping the 35 organisms by known trophic capacity for photosynthesis (14 out of 35 organisms), phagocytosis (16 out of 35 organisms), and prototrophy (21 out of 35 organisms). Note that trophic capabilities are not exclusive, organisms could fit into more than one group; see Burns et al. for grouping details [23; Supplementary Table 1]. Enrichment was determined by a significant proportion test [41]. Genes enriched for a trophic capability were clustered by gene ontology (GO) biological processes, and those GO clusters were summarized for each organism using a weighted scoring scheme. The set of the most predictive GO categories was determined using the Boruta algorithm for feature selection [42]. Predictions are called using a probability neural network, trained using the known trophic capabilities of the 35 training genomes, which outputs a numerical probability that a new organism will exhibit each capability [43].

Regarding green algae, the tool used three chlorophycean green algal genomes during training, scoring as photo-autotrophs, *Chlamydomonas reinhardtii*, *Chlorella variabilis*, and *Volvox carteri*, as well as one prasinophyte, *Cymbomonas tetramitiformis*, scoring as a phago-mixotroph. The prediction outputs were visualized by mapping four dimensions with three degrees of freedom (phagocytosis, photosynthesis, prototrophy, and a fourth dependent dimension for absence of each trophic mode) into color space using scripts modified from the R package “pavo” [44] and projecting the 4D data onto a 2D circular Mollweide projection as described in Jimenez et al. [45].

### Statistics

One-tailed Student’s *t*-tests were used to identify significant differences between average per_Δ_ values in CT treatments and those in CT-FLB + PFA treatments. Shapiro–Wilk tests were performed to confirm the normality of per_Δ_ values for each treatment. *F*-tests were performed to evaluate the homogeneity of variance between per_Δ_ values for CT and CT-FLB + PFA treatments for each strain. One-tailed Welch’s *t*-tests were used in instances where the assumption of homogeneity of variance was not met. A significance threshold of *p* ≤ 0.05 was used for all tests. All averages are reported ± standard deviation. All analyses were performed in R Studio [46].

## Results

### Feeding experiments measured by flow cytometry

**Fig. 2:**
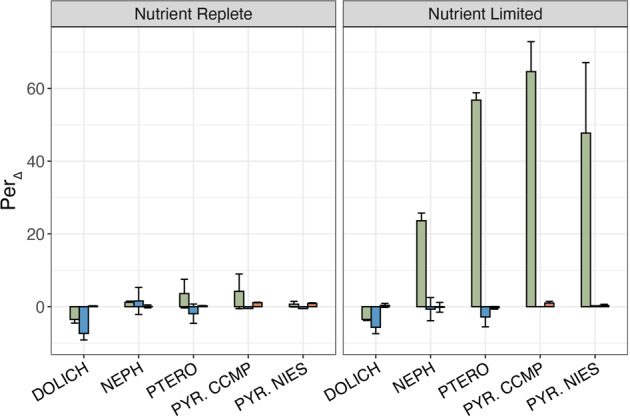
Values for per_Δ_ for algae fed with CT-FLB (green), DTAF- FLB (blue), or CT-FLB + PFA (red). The left plot corresponds to nutrient-replete treatments and the right plot corresponds to nutrient-limited treatments. DOLICH *Dolichomastix tenuilepis*, NEPH *Nephroselmis pyriformis,* PTERO *Pterosperma cristatum*, PYR. CCMP *Pyramimonas parkeae* CCMP726, PYR. NIES *Pryamimonas parkeae* NIES254. Error bars correspond to standard deviation of results for triplicate subsamples of a single culture flask. Negative results for *D. tenuilepis* may be attributed to its low ingestion rate plus photobleaching of extracellular fluorophores over the incubation period (See Supplementary Fig. 4).

Results of cytometry feeding experiments are summarized in Fig. 2. The average per_Δ_ value in the CT-FLB + PFA samples (i.e., PFA-killed, negative controls) across all strains tested was 0.4 ± 0.70% (*n* = 30) after 3 h of incubation. The average per_Δ_ value in samples inoculated with heat-killed prey bacteria (DTAF-FLB) across all strains tested was −2.6 ± 3.3% (*n* = 22). The average per_Δ_ value for nutrient-replete treatments inoculated with CT-FLB was 4.3 ± 10.2%. Average per_Δ_ values for nutrient-limited treatments inoculated with CT-FLB were 23.6 ± 2.10% for *N. pyriformis*; 56.79 ± 2.00% for *P. cristatum*; 64.6 ± 8.23% for *P. parkeae* CCMP726; 47.7 ± 19.38% for *P. parkeae* NIES254; and −3.5 ± 0.29% for *D. tenuilepis*. The negative values for *D. tenuilepis*, which nonetheless was observed to ingest CT-FLB by epifluorescence microscopy, may be attributable to photobleaching of background fluorescence over the course of the incubation (Supplementary Fig. 4). Per_Δ_ values were significantly greater in nutrient-limited treatments inoculated with CT compared to those inoculated with CT-FLB + PFA for *N. pyriformis* (Student’s *t*-test, *p* = 0.0001), *P. cristatum* (Welch’s *t*-test, *p* = 0.0002), *P. parkeae* CCMP726 (Welch’s *t*-test, *p* = 0.007), and *P. parkeae* NIES254 (Welch’s *t*-test, *p* = 0.02). Per_Δ_ values for treatments inoculated with CT were not significantly different compared to per_Δ_ values for those inoculated with CT-FLB + PFA in nutrient-limited *D. tenuilepis* treatments, nor in any nutrient-replete treatments. There were no significant changes in the average green fluorescence of FLB between t_0_ and t_3_ for CT-FLB, DTAF-FLB, or CT-FLB + PFA (Student’s *t*-test, *p* > 0.05 for all comparisons). Therefore, feeding results were assumed to not have been biased by changes in the fluorescence of FLB over the course of incubations.

### Feeding experiments observed by microscopy

**Fig. 3:**
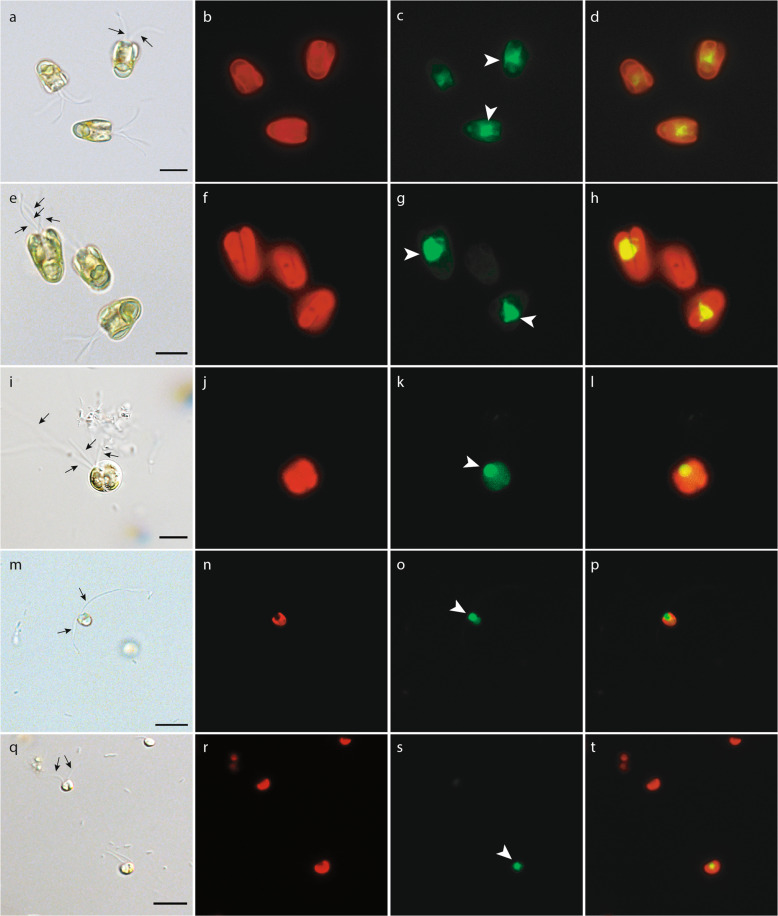
Epifluorescence microscopic observation of bacterivory in green algae. Light microscopy images of *Pyramimonas parkeae* NIES254 (**a**–**d**), *Pyramimonas parkeae* CCMP726 (**d**–**h**), *Pterosperma cristatum* NIES626 (**i**–**l**), *Nephroselmis pyriformis* RCC618 (**m**–**p**), and *Dolichomastix tenuilepis* CCMP3274 (**q**–**t**). The prasinophyte cells were fed with CT-FLB. The characteristic shape of the cells with their flagella (black arrows) were captured with the differential interference contrast optic (**a**, **e**, **i**, **m**, **q**). Red shows autofluorescence from the chloroplasts (**b**, **f**, **j**, **n**, **r**). The green spots localized centrally or close to the base of the flagella (white arrow heads), observed with FITC fluorescence, indicate ingestion of fluorescently labeled prey (**c**, **g**, **k**, **o**, **s**). An overlay of FITC and chlorophyll fluorescence images indicates the relative localization of the feeding compartment within the cells (**d**, **h**, **l**, **p**, **t**). Scale bars: 10 µm. See Supplementary Figs. 5–9 for additional images.

Epifluorescence microscopic observation of algal cultures fed with CT-FLB revealed positive feeding signals in all five tested strains, even *D. tenuilepis* (Fig. 3; Supplementary Figs. 5–9), but no evidence of ingestion was observed with the DTAF-FLB (not shown). One or two bright green fluorescent spheres were clearly visible in cells of the two *P. parkeae* strains (Fig. 3a–c for NIES254; Fig. 3d–f for CCMP726). When *P. cristatum* was inoculated with CT-FLB, we observed a fluorescing sphere that was notably brighter and bigger than the naturally green autofluorescent spot of unknown origin (Fig. 3g–i). Some *P. cristatum* cells exhibited two fluorescent spheres indicative of feeding compartments (not shown). Ingestion of CT-FLB was also observed for *N. pyriformis* (Fig. 3j–l) and *D. tenuilepis* by epifluorescence microscopy (Fig. 3m–o). Confocal microscopic observations (Supplementary Materials SM2) indicated that the green fluorescence was located centrally, lodged between the lobes of the chloroplast and directly under the flagellar pit in *P. parkeae* NIES254 (Supplementary Fig. 10a) and at or under the base of the flagellar pit for the other green algal strains (Supplementary Fig. 10b–d). In contrast to feeding experiments with CT-FLB, we did not find a clear indication of magnetic bead ingestion by any of the algal strains (Supplementary Materials SM3).

### Predictions of phago-mixotrophy using a gene-based model

**Fig. 4:**
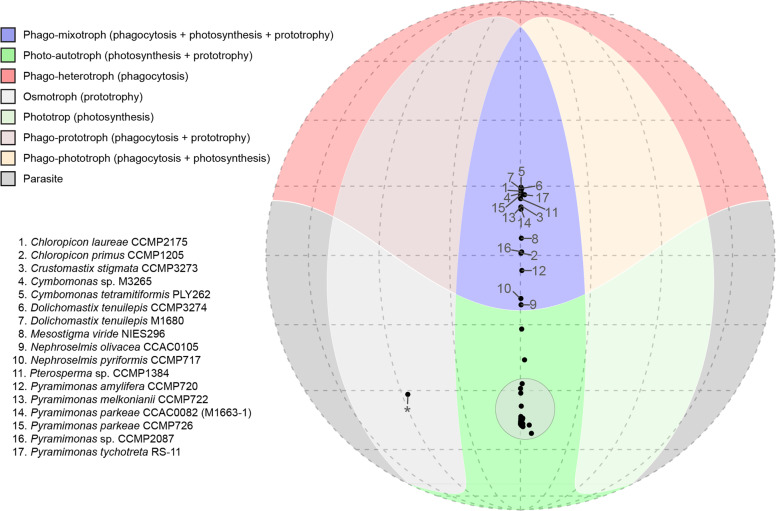
Mollweide projection showing the 4D positioning of the combination of predictions for phagocyte, prototrophy, and photosynthesis. Dark-colored regions indicate overlapping areas where individual predictions were >0.50. Gray regions indicate areas where all three predictions were <0.50. Trophic mode categories are based on the combinations of predicted capabilities. Each black dot indicates one of the 90 strains tested with the gene-based model. Numbered strains indicate the green algal strains predicted to be phago-mixotrophic. The vast majority of the surveyed taxa fall within the shaded circle (70 in total) in the green photo-autotroph region. An asterisk indicates the prediction for *Polytomella parva* SAG63-3. Note that of these 90 algae, *Cymbomonas tinamiformes* PLY262, *Chlamydomonas reinhardtii*, and *Volvox carteri* were used in the training sets, coded as a phago-mixotroph or a photo-autotroph, during the formation of the in silico prediction model [22]. Refer to Supplementary Table 1 for more detailed information.

The predictive model identified 17 out of 90 green algal strains as phago-mixotrophic while most of the green algae clustered as photo-autotrophs and one strain was predicted to be a strict osmotroph, a prototroph that lacks both photosynthesis and phagocytosis (Fig. 4; Supplementary Fig. 11). The prediction scores represent the probability that a given organism is capable of the function being tested on a scale of 0 to 1. A probability greater than 0.5 is interpreted as evidence that the organism can carry out a given function, with higher scores suggesting increased confidence in the functional prediction. Prediction scores for all 90 strains are provided in full detail in Supplementary Table 1. All nine strains of Pyramimonadophyceae surveyed, representing the three genera *Cymbomonas, Pterosperma*, and *Pyramimonas*, were predicted to be phago-mixotrophs with high phagocyte scores (>0.64). In addition, the two Nephroselmidophyceae strains tested in the model also consistently showed high phagocyte prediction scores, albeit scoring lower than the phago-mixotrophic Pyramimonadophyceae, with 0.519 for *Nephroselmis olivaceae* CCAC0105 and 0.542 for *N. pyriformis* CCMP717. Within the Mamiellophyceae, only two species scored high on phagocyte predictions: *D. tenuilepis* CCMP3274 and M1680 (both with a score greater than 0.9) and *Crustomastix stigmatica* CCMP3273. The other mamiellophyceans with the exception of two *Mantoniella* strains were predicted as non-phagotrophs with scores below 0.05. *Mantoniella antarctica* and *M. beaufortii* received prediction scores of 0.43 and 0.22, respectively. In addition, two chloropicophycean strains, *Chloropicon primus* CCMP1205 and *Chloropicon laureae* CCMP2175 were predicted as phago-mixotrophs, scoring 0.707 and 0.979, respectively, as well as the basal streptophyte *Mesostigma viride* NIES296 with 0.764. Interestingly, other strains of *Chloropicon* and *Mesostigma* scored comparatively lower in phagocyte predictions, e.g., *Chloropicon mariensis* RCC998 (0.002) and *C. roscoffensis* CCMP1998, RCC1871, and RCC2335 (0.109, 0.002, and 0.007, respectively), and *Mesostigma viride* SAG50-1 (0.006) and NIES995 (0.323). All of the strains mentioned above consistently scored high for photosynthesis and prototrophy predictions (Supplementary Table 1). As expected, *Polytomella parva* SAG63-3, a colorless freshwater flagellate with a vestigial plastid [47, 48], was predicted as an osmotroph, with a high prototroph score (0.967) but low phagocyte (0.002) and phototroph scores (0.157).

## Discussion

**Fig. 5:**
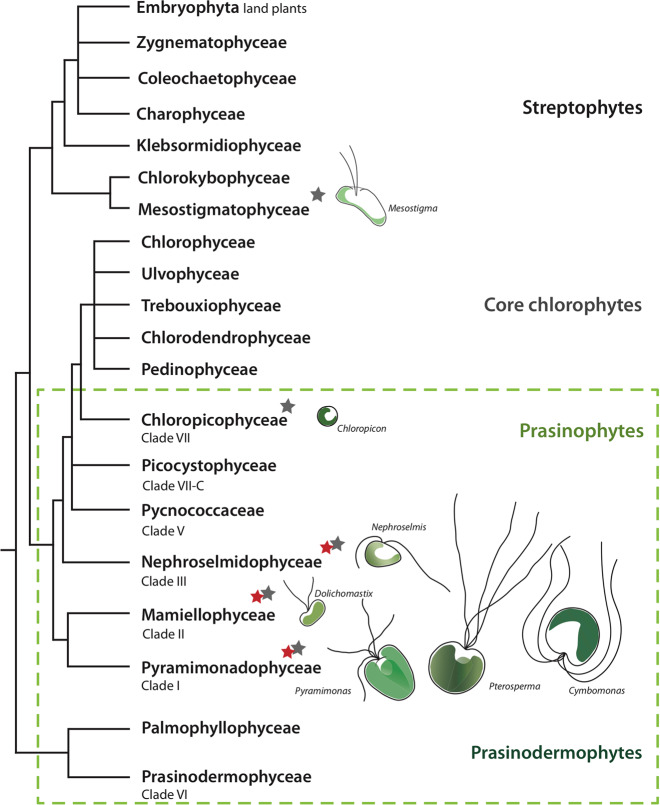
Summary diagram of phago-mixotrophy detected among the green algae. Details of phylogenetic relationships for the core chlorophytes and prasinophytes, in addition to branching phylogenies for streptophytes, are based on previous studies [51, 91–93]. Red stars indicate the clades with experimentally validated mixotrophic representatives, including *Cymbomonas* [20]; *Dolichomastix* [this study]; *Nephroselmis* [14, this study]; *Pyramimonas* [16, 48, this study]; and *Pterosperma* [this study]. Gray stars indicate the clades with representatives, such as *Crustomastix* and some strains of *Chloropicon* and *Mesostigma*, that are predicted as phagotrophs by our gene-based analyses.

Our experimental results demonstrate the occurrence of bacterivory across all of the prasinophyte strains tested, spanning three prasinophyte groups: the Pyramimonadophyceae, the Nephroselmidophyceae, and the Mamiellophyceae. The strains, chosen for the feeding experiments in this study, sample a deeper range of diversity within the prasinophytes (Fig. 5). Among the Pyramimonadophyceae, we chose *P. parkeae* NIES254 and CCMP726, two strains isolated from different sides of the Pacific Ocean, off the coast of Japan and Monterey Bay, respectively. *P. cristatum* NIES626, also isolated in Japan, is the first species of this genus to be confirmed as a bacterivore thus confirming the earlier speculations based on ultrastructural studies [20, 21, 24]. *N. pyriformis* RCC618, which represents the Nephroselmidophyceae (clade III), was isolated from the Gulf of Mexico at the Galveston Channel station and is a different strain from the species investigated for bacterivory by Anderson et al. [6], K-0557. *D. tenuilepis* CCMP3274, isolated from the Gulf of Naples, Mediterranean Sea, belongs to the Mamiellophyceae (clade II).

Results from microscopy revealed a green fluorescing compartment within the cells of all five algal strains following inoculation with CT-FLB. Flow cytometry detected significant increases in per_Δ_ for all nutrient-limited treatments inoculated with CT-FLB compared to CT-FLB + PFA controls, with the exception of *D. tenuilepis*. No significant differences in per_Δ_ values were observed for nutrient-replete treatments inoculated with CT-FLB compared to CT-FLB + PFA controls. It seems unlikely that significant increases in per_Δ_ were due to factors other than the ingestion of fluorescently labeled prey. The immediate increase in green fluorescence signal observed for all strains with flow cytometry, presumably due to excess extracellular fluorophores in the FLB cell suspensions, was accounted for in per_Δ_ estimates. However, given the limited photostability of fluorescein dyes, such as CellTracker CMFDA and DTAF [49], exposure of extracellular fluorophores to light during incubation may have diminished the intensity of background fluorescence over time. Hence, the fluorescence measured likely reflected both increases in algal cell fluorescence due to the ingestion of labeled prey and decreases in background fluorescence due to the photobleaching of extracellular fluorophores (Supplementary Fig. 4a). In cases where ingestion rates are low, this phenomenon may result in net negative per_Δ_ values (Supplementary Fig. 4b), as were observed in some nutrient-replete treatments and across all *D. tenuilepis* experiments.

Green algae, including prasinophytes, were traditionally considered as strict phototrophs [50, 51], despite several earlier studies that suggested the potential phagotrophic behavior in some prasinophytes [13, 21, 52–55]. More recently, there has been a resurgence of research on bacterivory by green algae, including *Cymbomonas tetramitiformis*, *Micromonas* sp. CCMP2099 [15 but see 45], *Pyramimonas tychotreta*, and *Mantoniella antarctica* [15], as well as *N. pyriformis* (K-0557) and *N. rotunda* (K-0556) [6]. The current study further expands the known extent to which bacterivory occurs among green algae. Our gene-based predictive model of phagocytosis, in particular, suggested a potential for bacterivory in several green algal genera, distributed in five distinct green algal sub-lineages (Fig. 4). The predictions themselves are not determinate; they use gene content from genomic or transcriptomic data to suggest the capacity for phagocytosis. They also do not specify a set of genes that an organism must have to be considered a phagocyte, but rather determine that an organism has a subset of characteristic genes consistent with the capacity for a trait. Therefore, the predictions are most powerful when combined with experimentation. Strains of *Dolichomastix, Nephroselmis, Pterosperma*, and *Pyramimonas* were identified as bacterivores both by in silico predictions and empirical evidence. Of the five strains that are newly confirmed to be phagotrophic by laboratory assays in this study, large-scale genetic data currently exist for *P. parkeae* CCMP726 and *Dolichomastix tenuilepis* CCMP3274. Therefore, we note that the gene-based model of trophic modes and experimental results concur at least for these two strains.

Furthermore, the model predicted three additional green algal taxa to be phago-mixotrophs, including *Chloropicon primus, Crustomastix stigmatica*, and *Mesostigma viride*. The dataset used for *Chloropicon primus* CCMP1205 is the first complete genome available for the recently characterized Chloropicophyceae [56]. This prediction is particularly surprising in that members of the Chloropicophyceae are small scale-less non-motile cells of 2–3 µm [57]. Nonetheless, the genome of *Chloropicon primus* contains genetic signatures for the formation of both scales and flagella [58]. Therefore, it is possible that this species has a previously undocumented flagellate life stage, where bacterivory may play a role in nutrition. *Crustomastix stigmatica* is small (3–4 µm long ×2 µm wide) and bears two flagella. Its cell body is not covered with scales, although hairy scales cover the flagella [59]. In addition, the prediction of phago-mixotrophy for the genome of the freshwater *M. viride* NIES296 [60], an early diverging streptophyte, supports the hypothesis that bacterivory was present in the common ancestor of Chloroplastida. In terms of characteristic genes in these species, WASH complex proteins, responsible for recycling of membrane proteins from phagosomes back to the cell surface [61], were present in all three datasets (Supplementary Table 2). A second phagocytotic hallmark, DNase II [62], is found in both *Chloropicon primus* and *Crustomastix stigmata*, but is absent from *M. viride* (Supplementary Table 2).

The model predictions provided contrasting predictions for strains of the same species or genus, such as *Mesostigma* or *Chloropicon*. One strain or species could be predicted to be phago-mixotrophic while another was positioned as photo-autotroph. The contrasting predictions for *Chloropicon* species can in part be seen with the WASH proteins where WASH complex subunits 1 and 4 are detected in all datasets, but subunits 2, 3, and 5 are only detected in the two strains that received a positive prediction for phagocytosis, *Chloropicon primus* CCMP1205 and *Chloropicon laureae* CCMP2175 (Supplementary Table 2). DNase II was detected in all *Chloropicon* strains (Supplementary Table 2), regardless of the prediction of phagocytotic capacity, emphasizing the combinatorial nature of the predictions; it is not possible to rely on any one gene or process to make a prediction about phagocytotic capacity. The differential detection of phagocytosis related proteins and resultant contrasting predictions, particularly between strains of the same species, may be attributed to the inherent bias of transcriptomic data in that they reflect protein repertoire expressed in particular physiological conditions. Alternatively, the contrasting interspecific predictions might reflect recent losses of bacterivory in some green algae. For *Mantoniella antarctica*, there is discordance between the model prediction and previous experimental results [63]. The contrasting claims are reconcilable when considering that the prediction score of 0.43 attained for this alga, abutting the phago-mixotroph threshold, is likely due to incompleteness of the transcriptome data. Indeed, previous predictions of an earlier annotation of this same MMTESP *Mantoniella antarctica* transcriptome provided a phagotroph score of 0.09 [45], emphasizing the sensitivity of the model to annotation methods. More experimental and sequencing efforts are needed to clarify these issues. Phago-mixotrophy is a spectrum [64] and our model provides avenues to develop new hypotheses on the genetic basis of contrasting mixotrophic behaviors among closely related taxa.

Synthesizing both empirical and in silico prediction data, it appears that phago-mixotrophy is broadly preserved among early diverging members of green algae. However, despite their recognized abundance in many marine regions [17, 19, 65–67], prasinophyte contribution to bacterivory remains conspicuously undetected in most environmental studies [5]. The global underestimation of prasinophyte bacterivory might hinge on the class they are represented by in a given environment. Chloropicophyceae tend to be prevalent in the open ocean, such as the oligotrophic South Pacific Subtropical Gyre, where they contribute significantly to primary production [19, 57]. Our in silico predictions for *Chloropicon primus* and *C. laureae* suggest that members of this class have the potential to feed on bacteria. Coastal prasinophytes are generally represented by the Mamiellophyceae [18, 67]. Our predictions showed phago-mixotrophic potential for some members, including *D. tenuilepis* and *Crustomastix stigmatica*, the former of which was also experimentally confirmed to ingest bacteria. However, whether or not other members like *Micromonas* CCMP2099 are capable of consuming bacteria remains debated [14, 45] and needs to be further investigated. In addition, the limited detection of prasinophyte bacterivory in situ may be attributable to the association of feeding with particular environmental conditions, even for a bacterivorous class, such as the Pyramimonadophyceae for which reports of bacterivory are more frequent from polar systems [13, 63].

It is also important to consider methodological sources of bias, such as the source of experimental prey. Many protist predators graze selectively [68–71], with prey size being an important factor. Natural assemblages of marine heterotrophic flagellates and ciliates tend to select larger, actively growing bacterial cells [72–76], with prey size in particular affecting the probability of encounter, the capacity for capture and the nutritional value of the prey [77–79]. Prey size might also affect the rate at which prey is digested and decomposed, preventing detection of feeding if ingested prey are digested faster than expected, or if they are egested quickly following ingestion [80–83]. In this study, we observed that *D. tenuilepis* did not ingest a relatively large bacterial prey (*Alteromonas macleodii*) that was successfully consumed by larger strains, including *P. cirstatum* NIES626 and *P. parkeae* NIES254 and CCMP726 (unpublished data).

In addition to the prey size, other factors also play roles in feeding selectivity, such as release of chemical stimuli, prey motility, biochemical composition, and cell surface compounds [80]. In addition, inert surrogate prey (plastic beads or heat-killed bacteria) have been reported to be less recognizable or ingestible by some protists [75, 80], such as the phago-mixotrophic *Prymnesium* [84] and *Dinobryon* [80, 84, 85]. Therefore, the apparent preference of the green algal strains for the live over the heat-killed FLBs that we observed in this study may be due to significantly reduced ingestion as a result of denaturing of cell structure and biochemistry in the course of the incubations at 60 °C, required for the DTAF labeling protocol. It may also be the case that binding of DTAF fluorophores to the cell surface prevents algal cells from recognizing the DTAF-FLBs as prey. Because cellTracker is a cytoplasmic stain, this would not be an issue in CT-FLB. Similar to DTAF-FLB, magnetic beads were not ingested by the green algae although the attachment of a bead to the area near the cell’s anterior was occasionally observed for *P. cirstatum* NIES626 (data not shown). In previous experiments with *P. cirstatum* NIES626, only limited feeding was observed on pHrodo^TM^ bacterial particles, which are chemically fixed, further demonstrating potential preference for live prey in the surveyed strains. While we were not able to confirm ingestion of DTAF-FLB or magnetic beads by any of the green algae, feeding on killed prey might have occurred at a frequency below the detection limits of the methods used in this study (Supplementary Materials SM4).

## Conclusions

Small mixotrophic protists enhance carbon transfer within the microbial loop [3, 86]. Indeed, as they feed on their competitors for inorganic nutrients these small mixotrophs compete with their own predators for prey biomass [87] and possibly constitute a more nutritious food source for higher trophic levels [88, 89]. In addition, mixotrophic protists exhibit versatile feeding behaviors [90], which likely affects the nature of the top-down control on prey communities and on carbon cycling. Our results highlight the prevalence of phago-mixotrophy among early diverging green algae and demonstrate the advantage of using live-stained FLB to assay bacterivory in mixotrophic algae. Contributions of green algal bacterivory to microbial community structure and biogeochemical cycling may have previously been underestimated and require further investigation. Additional research is also needed to experimentally validate in silico predictions of phago-mixotrophy in green algae and to characterize the cell structural basis of green algal bacterivory as well as its regulation in response to environmental change.

## Supplementary information

Supp Materials & Figures

Supp Table 1

Supp Table 2
