# Supplementary material for: Experimental identification and in silico prediction of bacterivory in green algae
Source: ISME J. 2021 Mar 2;15(7):1987–2000. doi: 10.1038/s41396-021-00899-w (PMC8245530; doi:10.1038/s41396-021-00899-w)
Supplement: Supplementary file 1 — Supp Materials & Figures [file 41396_2021_899_MOESM1_ESM.pdf]

Supplementary materials

SM1.1 ***Prey viability tests method***

To test the effect of the Cell Tracker CMFDA labeling protocol on the viability of *Pelagibaca bermudensis*, three dilutions of the bacterial culture were prepared at late exponential phase to final cell abundances of ~ 1 x 10^9 cells mL^{-1} in 1 mL of ASW. One dilution was maintained at normal growth temperatures of 25° C; one dilution was incubated for three hours at 37° C, washed three times in ASW, then stored at 4° C; one dilution was inoculated with CT, incubated for three hours at 37° C, washed three times in ASW, then stored overnight at 4° C. Following these treatments, 100 mL aliquots from each cell suspension were used to inoculate triplicate tubes of enriched growth medium (ESW; 0.2% glucose, 0.5% yeast extract, w/v in artificial seawater). Cultures were then incubated for three days at 25° C, with absorbance of cultures at 670 nm being measured via photospectrometry at 24-hour intervals. Triplicate culture tubes of ESW, not inoculated with bacteria, were incubated at 25° C as a negative control. Growth rates (Δ absorbance day^{-1}) were estimated for each experimental replicate by solving the exponential growth formula based on absorbance measurements after three days' incubation:

$$\frac{ln\,\frac{x_3}{x_0}}{3} = k$$

Where x_0 corresponds to the culture absorbance at t_0, x_3 corresponds to the culture absorbance at t_3, and k corresponds to the growth rate of the culture.

SM1.2 ***Prey viability tests results***

Average growth rates for *P. bermudensis* cultures started from cell suspensions incubated at normal growth temperatures were 0.27 ± 0.02. Average growth rates for *P. bermudensis* cultures (started from cell suspensions inoculated with CellTracker, incubated three hours at 37° C, and then stored overnight at 4° C) were 0.32 ± 0.02. Average growth rates for *P. bermudensis* cultures started from cell suspensions incubated three hours at 45° C were 0.09 ± 0.095. Growth rates for *P. bermudensis* cultures started from cell suspensions inoculated with CellTracker were significantly greater than those started from cell suspensions maintained at normal growth temperatures (Student's t-test, $p = 0.03$, $n = 3$). Growth rates for *P. bermudensis* cultures started from cell suspensions incubated at 45° C were not significantly different than growth rates for negative controls (Student's t-test, $p = 0.15$, $n = 3$).

SM2 ***Confocal microscopic examination of bacterivory by green algae***

Confocal microscopy was conducted on the same treatments as for epifluorescence using a LSM710 (Carl Zeiss, Germany) microscope with an argon/2 laser to excite at 488 nm and optical sections were acquired at 517 nm and 680 nm for CellTracker Green or DTAF (FLB) and chlorophyll autofluorescence, respectively, to detect presence of ingested bacterial prey in the phagotrophic algal cells. Images were collected with a mounted digital microscope camera AxioCam.

SM3 ***Feeding experiments using magnetic beads***

The chosen strains of green algae were also subjected to feeding experiments with magnetic beads. We selected magnetic beads for ingestion experiment with the original intention to use it for green algal phagosome isolation and proteomics characterization in a follow-up study [1]. Experiments were carried out with 0.5 µm Mono Mag Carboxylic Acid Beads (Ocean Nanotech, San Diego, California) or 1 µm Dynabeads MyOne Carboxylic Acid (ThermoFisher Scientific). Algae at different growth phases were pelleted, re-suspended in an Eppendorf tube, given sufficient time (2 days) to regrow flagella and then fed with magnetic beads. Prior to inoculation, the magnetic beads were washed 2× in filter-sterilized ASW and re-suspended in a thin-walled glass vial for 5-min sonication (Branson 200 Ultrasonic Cleaner) in order to break apart bead aggregates. Beads were then added to the cells at a concentration of 1 µL beads per mL culture. Negative control samples received the same volume of sterile ASW. The obligate heterotrophic flagellate *Palpitomonas bilix* [2] was used as a positive control. After adding the beads or ASW, the mixture was incubated for 30 min on a hula mixer to stir the beads. In parallel, aliquots of the cultures were concentrated with the magnetic beads, to potentially increase encountering beads and thus increase consumption, by pelleting the cells after bead addition in a secondary Eppendorf at 500 g. Cells were observed under a Zeiss Axiovert 100M microscope, after 30-90 min since the start of incubation.

SM4 ***Detection limits and sources of variability in cytometry results***

Detection of feeding in algal strains with low ingestion rates may have been limited by changes in fluorescence due to factors other than ingestion alone. Interpreting average per_{fed} values in

PFA controls as a blank value, the ingestion detection limit three hours after inoculation with cell tracker is approximately 1.5 % (corresponding to the mean increase in per_{fed} observed in CT+PFA treatments plus two times the standard error). Additionally, activated Cell Tracker dye was commonly observed to accumulate in FLB suspension following the labeling protocol, presumably due to activation of dye by extracellular enzymes [3]. The presence of activated dye was repeatedly observed to cause elevated background fluorescence both in cytometry and microscopy samples, even following the introduction of additional wash steps and the storage of FLB suspensions at 4°C. While initial background fluorescence was accounted for in cytometry experiments by taking measurements of algal cells immediately following inoculation with FLB, interpretation of results obtained when working with CellTracker could be complicated due to photobleaching of extracellular fluorophores over the course of incubations. Especially in strains with low feeding rates, such as *D. tenuilepis*, increases in algal cell fluorescence measured via flow cytometry may in fact reflect a net change, capturing the sum of 1) increases in fluorescence due to ingestion of labeled prey and 2) decreases in fluorescence due to photobleaching. This may explain the decrease in algal cell fluorescence over time in the case of *D. tenuilepis*, as well as the occasional observation of feeding in CT-FLB inoculated nutrient replete cultures via microscopy but not via flow cytometry. Although it is possible to eliminate effects of background fluorescence in cytometry experiments by reducing the sensitivity of the green fluorescence photomultiplier tubes, it is unclear what criteria should be used to determine the magnitude of this adjustment across different strains. Therefore, the same sensitivity settings were used for all experiments in this study.

References

1. Lönnbro P, Nordenfelt P, Tapper H. Isolation of bacteria-containing phagosomes by magnetic selection. *BMC Cell Biol* 2008; **9**: 35.

2. Yabuki A, Inagaki Y, Ishida K. *Palpitomonas bilix* gen. et sp. nov.: A Novel Deep-branching Heterotroph Possibly Related to Archaeplastida or Hacrobia. *Protist* 2010; **161**: 523–538.

3. First MR, Park NY, Berrang ME, Meinersmann RJ, Bernhard JM, Gast RJ, et al. Ciliate ingestion and digestion: Flow cytometric measurements and regrowth of a digestion-resistant *Campylobacter jejuni*. *J Eukaryot Microbiol* 2012; **59**: 12–19.

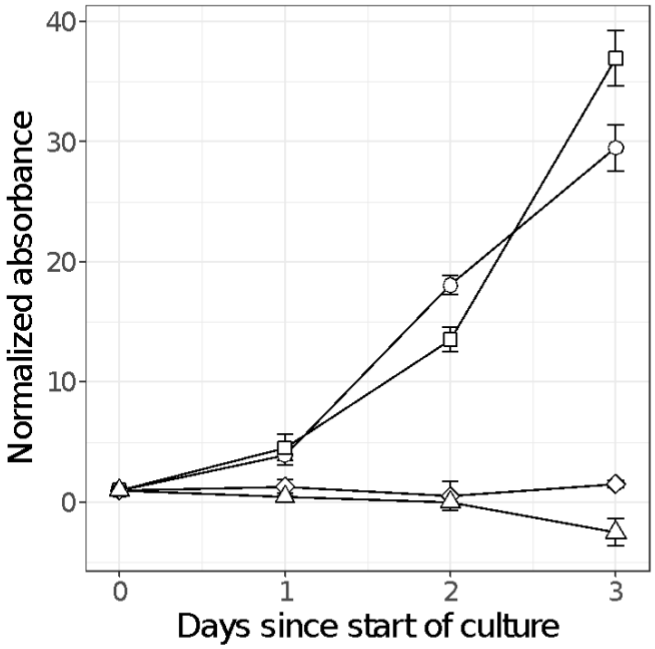

Supplementary figure 1: Culture absorbance over three days in *Pelagibaca bermudensis* viability tests. Square markers correspond to cultures started from *P. bermudensis* cell suspensions following labeling protocol and overnight storage at 4° C; diamond markers correspond to cultures started from *P. bermudensis* cell suspensions incubated at 45° C; circle markers correspond to cultures started from *P. bermudensis* cell suspensions maintained under normal growth conditions; triangle markers correspond to absorbance of growth medium alone.

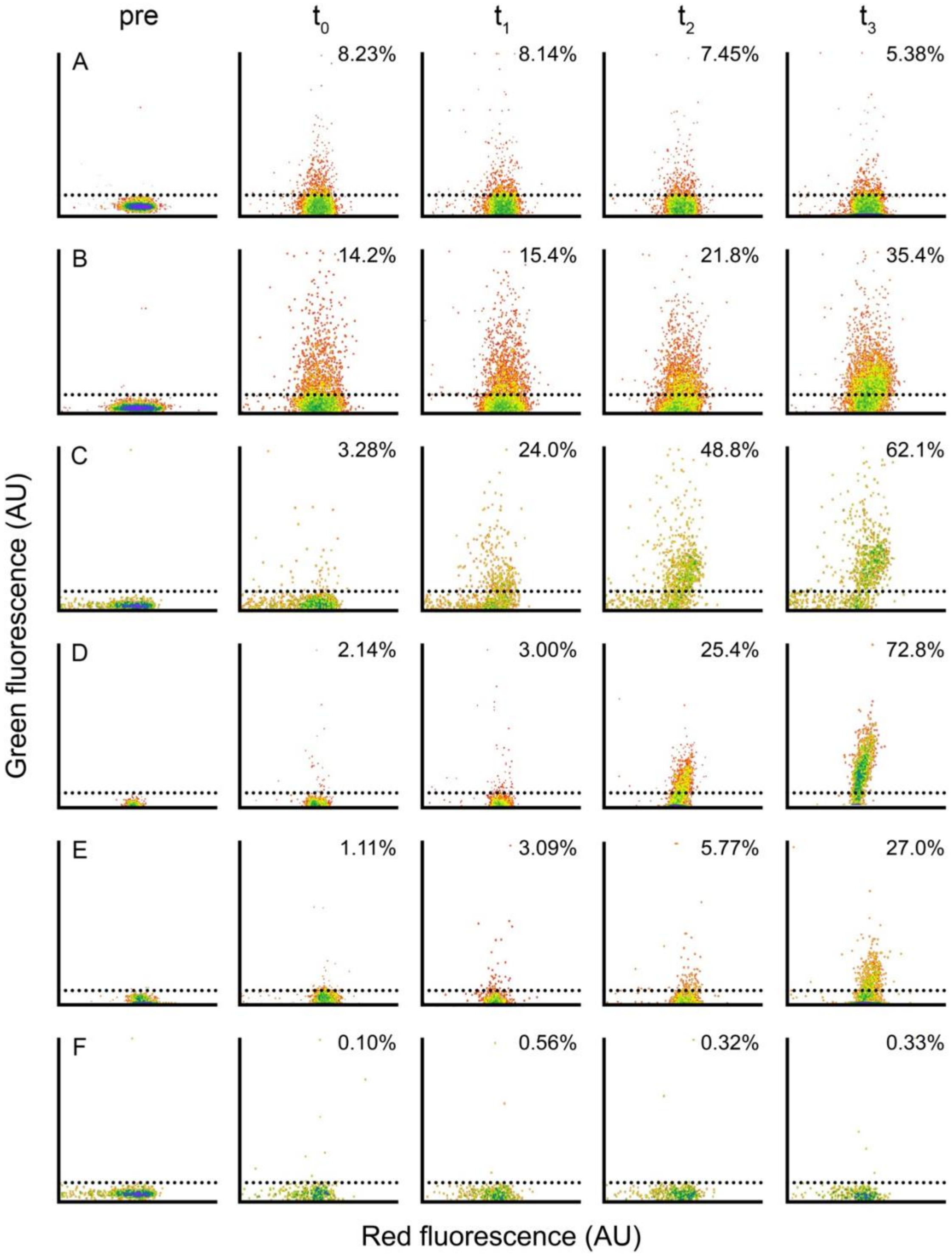
pre
t_0
t_1
t_2
t_3
A
8.23%
8.14%
7.45%
5.38%
B
14.2%
15.4%
21.8%
35.4%
C
3.28%
24.0%
48.8%
62.1%
D
2.14%
3.00%
25.4%
72.8%
E
1.11%
3.09%
5.77%
27.0%
F
0.10%
0.56%
0.32%
0.33%
Green fluorescence (AU)
Red fluorescence (AU)

Supplementary figure 2: Representative cytometry results for nutrient-limited treatments inoculated with CT-FLB for *Dolichomastix tenuilepis* (A), *Nephroselmis pyriformis* (B), *Pterosperma cristatum* (C), *Pyramimonas parkeae* CCMP726 (D), and *Pyramimonas parkeae* NIES 254 (E). Representative cytometry results for nutrient-limited treatments inoculated with CT-FLB+PFA for *Pterosperma cristatum* (F). Cytograms correspond to cytometry measurements made prior to inoculation with CT-FLB (pre), or to cytometry measurements made 0 to 3 hours following inoculation with CT-FLB (t_0 – t_3). Log-transformed red fluorescence plotted on the X axis in arbitrary units (AU); log-transformed green fluorescence plotted on the y axis in arbitrary units. For clarity, all cytograms were scaled to fill the plotting area and centered on the x axis. Dashed line indicates the position of GF_{prior}.

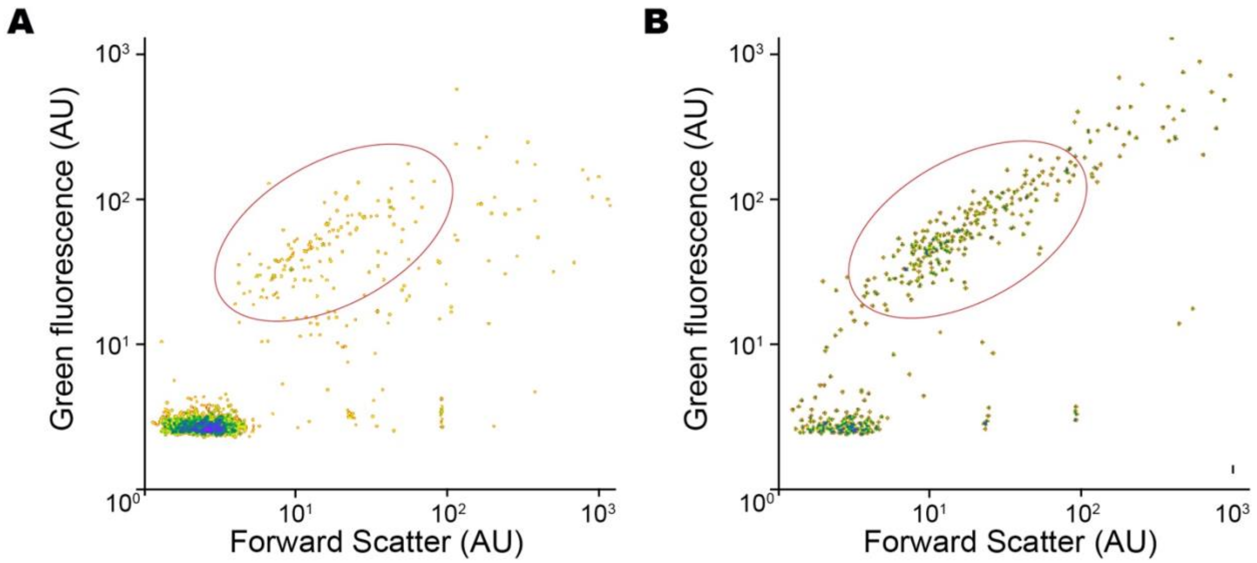

Supplementary figure 3: Cytograms corresponding to representative dilutions of CT-FLB (A) and DTAF-FLB (B). Events plotted based on log-transformed forward scatter (arbitrary units; AU) and log-transformed green fluorescence. Red ellipses highlight populations of labeled bacteria. Events in lower left corner of cytograms correspond to electronic noise. Events not plotted below 2.5 AU on y axis due to green fluorescence detector threshold settings.

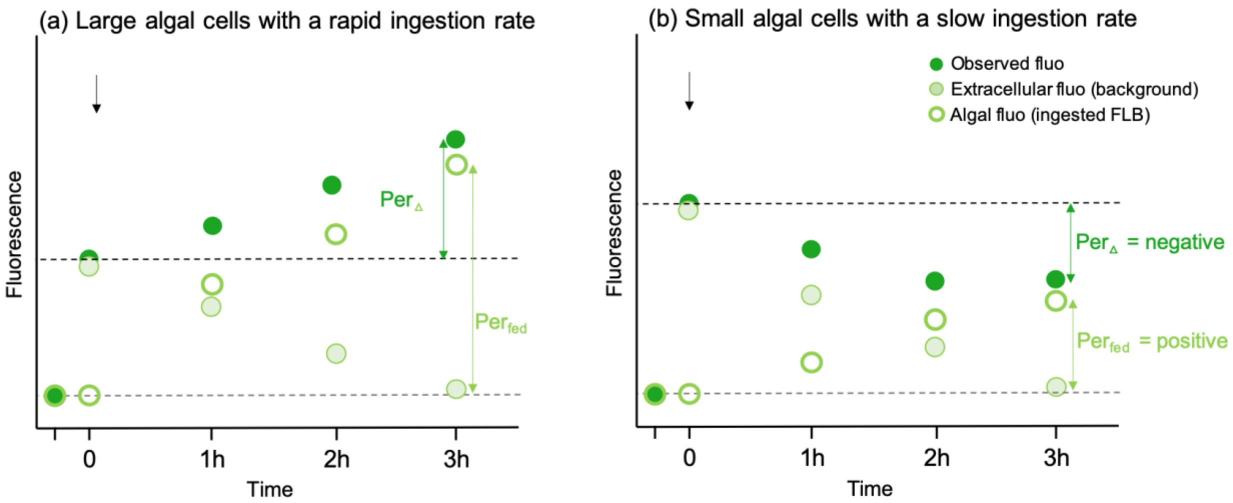

Supplementary figure 4: Conceptual representation of sources of underestimation in cytometry results of feeding experiments. The dark green dots represent the fluorescence observed with flow cytometry, which is the sum of the background fluorescence (light green dots) caused by extracellular fluorophores and the algal fluorescence (empty green circles) caused by ingestion of FLB. This conceptual model shows two contrasting trends in observed fluorescence over time as algal cells ingest the FLB and the extracellular fluorophores are bleached. For large algal cells (a), the bleaching of the initial background fluorescence leads to an underestimation of the percent feeding cells ($per_{\Delta} < per_{fed}$). For small cells (b) a greater initial background fluorescence and slower ingestion rates lead to an observed net decrease in fluorescence, masking the actual increase in algal fluorescence by ingestion ($per_{\Delta} < 0$; $per_{fed} > 0$).

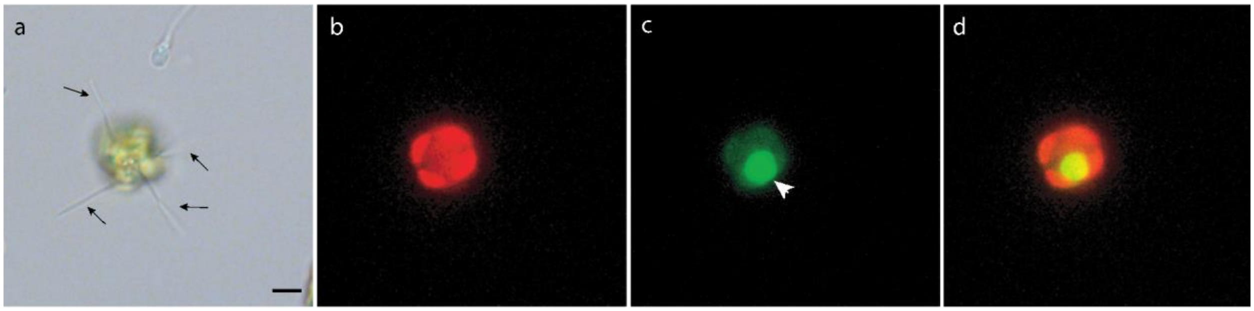

Supplementary figure 5: Additional light microscopy images of *Pyramimonas parkeae* NIES254. The prasinophyte cells were fed with CT-FLB. From left to right: differential interference contrast (DIC)(a), chlorophyll fluorescence channel (b), FITC fluorescence channel (c), and an overlay of chlorophyll and FITC fluorescence images (d). Four flagella of the alga are indicated with arrows (a). The compartment where ingested CT-FLB accumulated is indicated with an arrowhead (C). Scale bar: 10 µm.

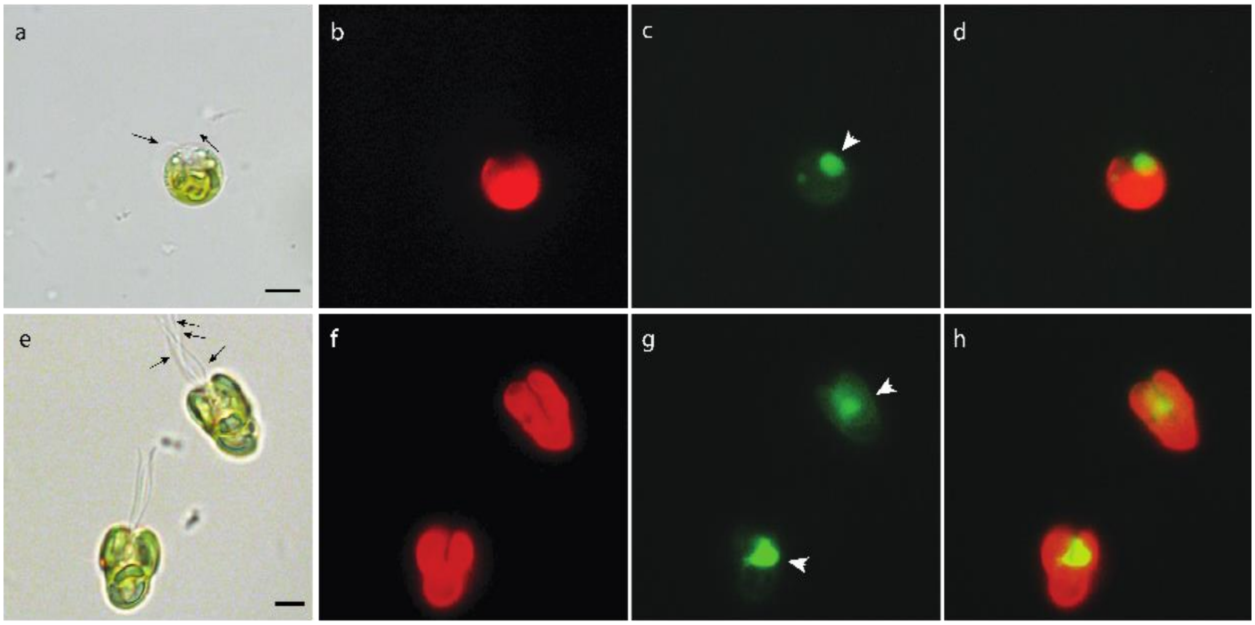

Supplementary figure 6: Additional light microscopy images of *Pyramimonas parkeae* CCMP726. The prasinophyte cells were fed with CT-FLB. From left to right: differential interference contrast (DIC) (a, e), chlorophyll fluorescence channel (b, f), FITC fluorescence channel (c, g), and an overlay of chlorophyll and FITC fluorescence images (d, h). The flagella of the alga are indicated with arrows (a, e). The intracellular compartment where the ingested CT-FLB accumulated is indicated with an arrowhead (c, g). Scale bars: 10 µm. Note that the cell presented in the top panels is more rounded in the posterior end compared to those presented below. This difference in morphology is likely due to the size of starch storage, which accumulates in the cell's posterior.

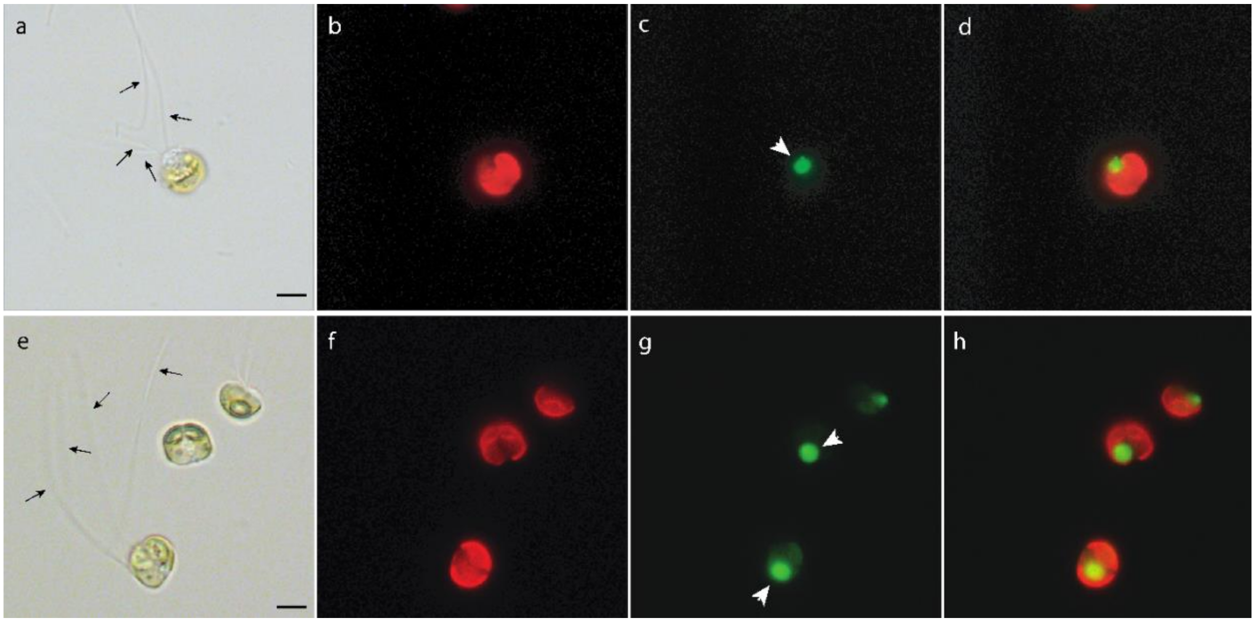

Supplementary figure 7: Additional light microscopy images of *Pterosperma cristatum* NIES626. The prasinophyte cells were fed with CT-FLB. From left to right: differential interference contrast (DIC) (a, e), chlorophyll fluorescence channel (b, f), FITC fluorescence channel (c, g), and an overlay of chlorophyll and FITC fluorescence images (d, h). Four flagella of the alga are indicated with arrows (a, e). The spherical compartment where the ingested CT-FLB accumulated is indicated with an arrowhead (c, g). Scale bars: 10 µm.

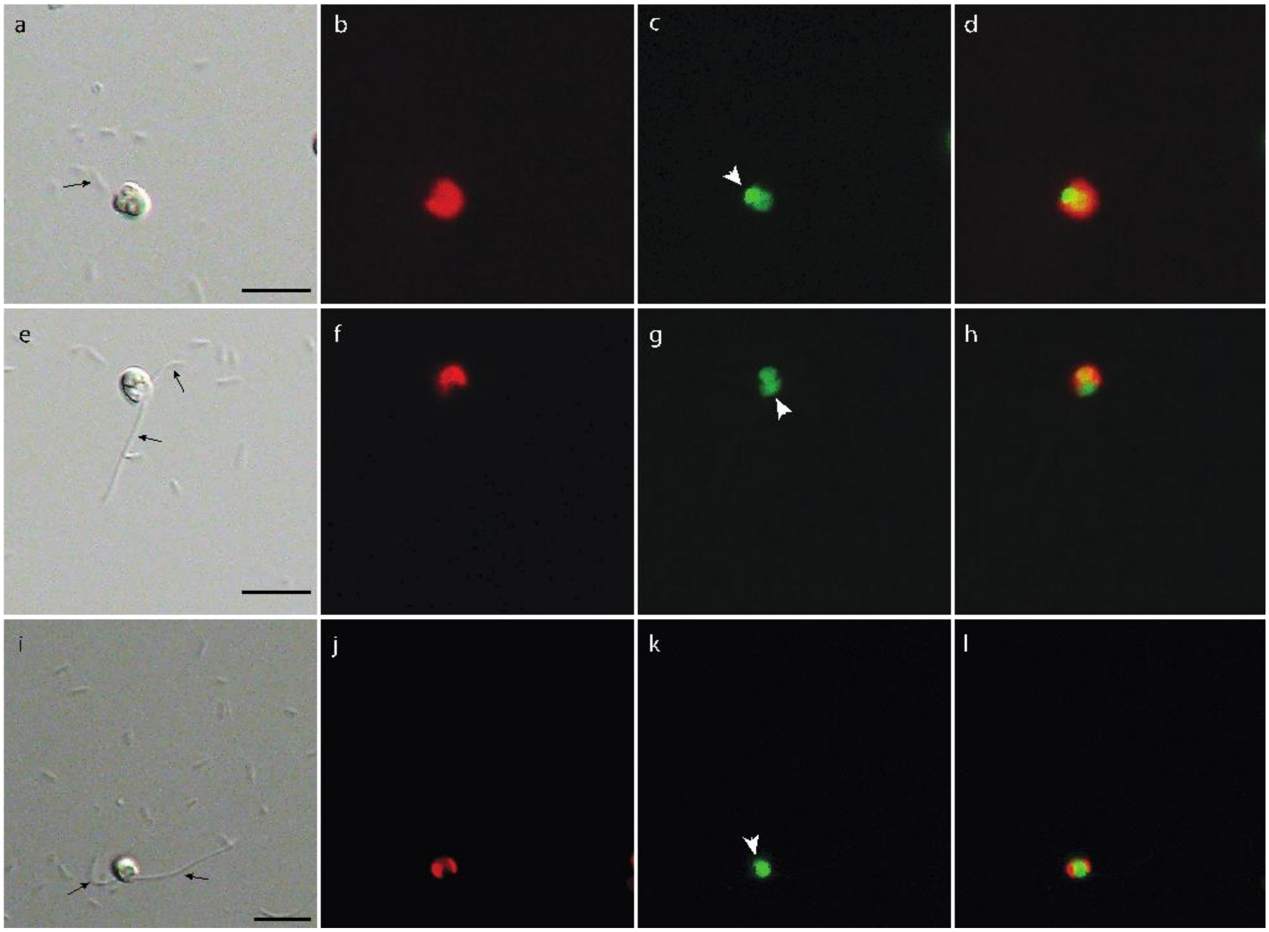

Supplementary figure 8: Additional light microscopy images of *Nephroselmis pyriformis* RCC618. The prasinophyte cells were fed with CT-FLB. From left to right: differential interference contrast (DIC) (a, e, i), chlorophyll fluorescence channel (b, f, j), FITC fluorescence channel (c, g, k), and an overlay of chlorophyll and FITC fluorescence images (d, h, l). The alga bears two flagella, indicated with arrows (a, e, i). The intracellular compartment where the ingested CT-FLB accumulated is indicated with an arrowhead (c, g, k). Scale bars: 10 µm.

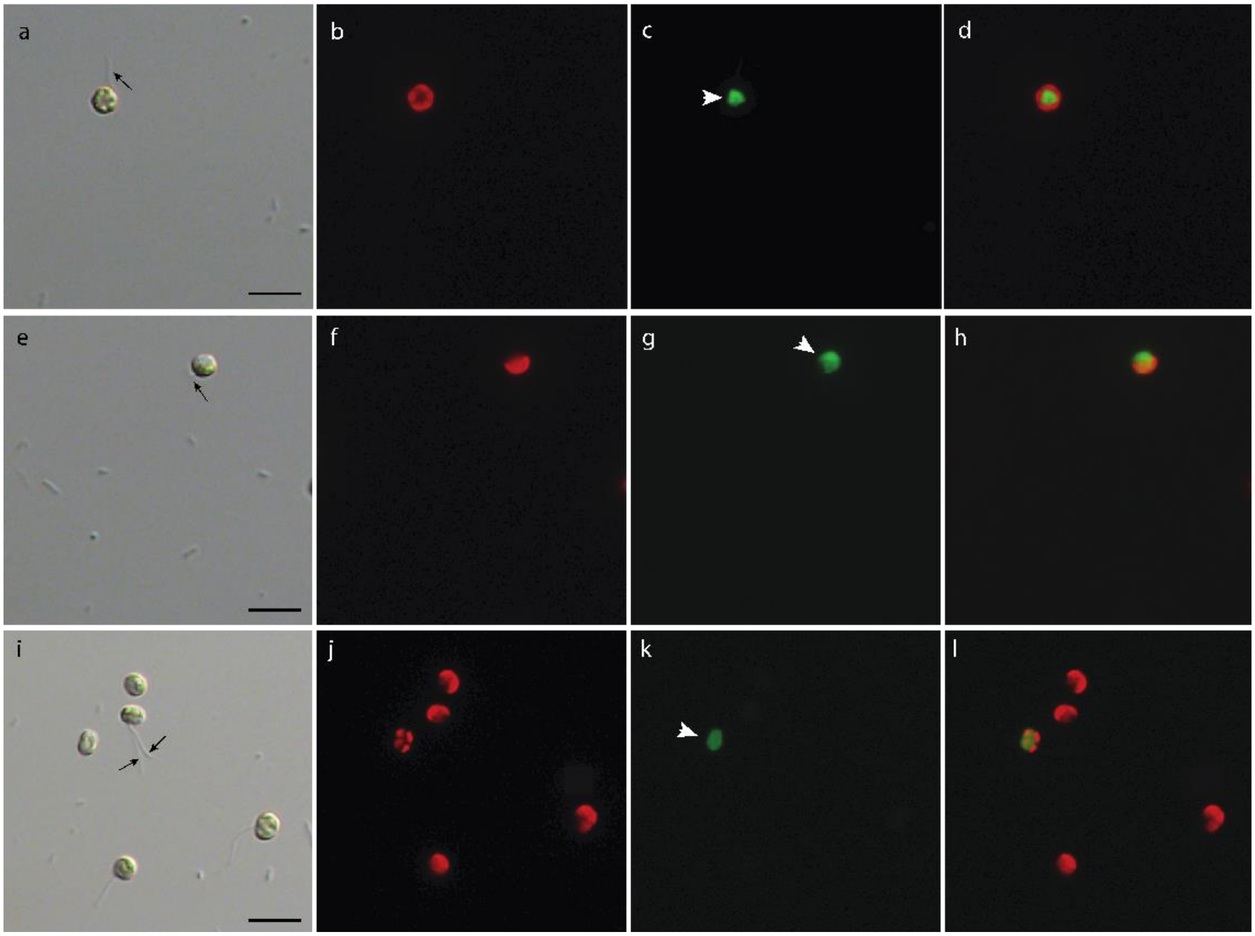

Supplementary figure 9: Additional light microscopy images of *Dolichomastix tenuilepis* CCMP3274. From left to right: differential interference contrast (DIC) (a, e, i), chlorophyll fluorescence channel (b, f, j), FITC fluorescence channel (c, g, k), and an overlay of chlorophyll and FITC fluorescence images (d, h, l). The alga bears two flagella, indicated with arrows (a, e, i). The intracellular compartment where the ingested CT-FLB accumulated is indicated with an arrowhead (c, g, k). Scale bars: 10 µm.

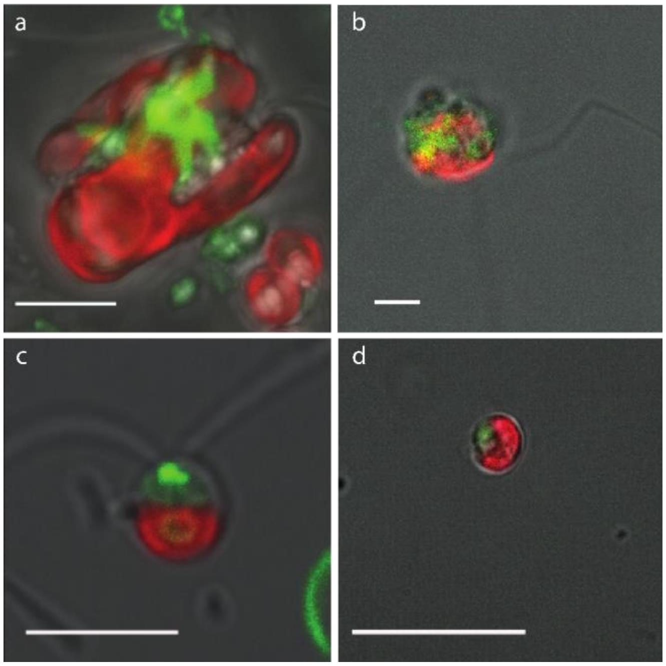

Supplementary figure 10: Images from confocal microscopy of *Pyramimonas parkeae* NIES254 (a), *Pterosperma cristatum* NIES626 (b), *Nephroselmis pyriformis* RCC618 (c) and *Dolichomastix tenuilepis* CCMP3274 (d) fed with a CT-labeled *Pelagibaca bermudensis* HTCC2601. Images acquired at 517 nm (for detecting the labeled bacteria) and 680 nm (for detecting the chloroplast compartment) were overlaid onto the corresponding bright-field image. Scale bars: 10 µm in (a) and (b), 5 µm in (c) and (d).

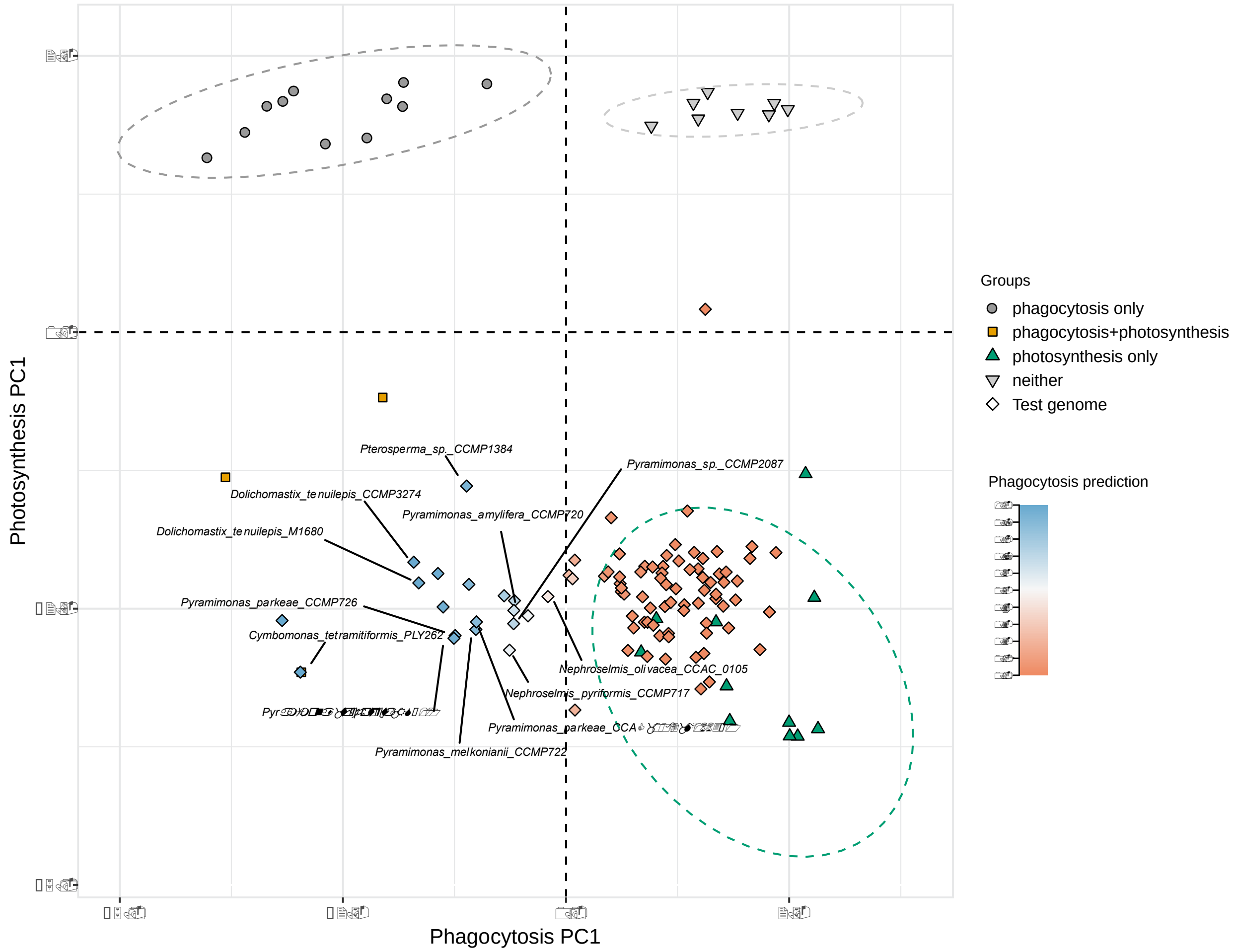

Supplementary figure 11: Principal component summary of phagocytosis and photosynthesis functions. This 2-dimensional projection represents the first principal components for phagocytosis functional category scores (x-axis) and photosynthesis functional category scores (y-axis). Reference genome placements in this plot are represented by grey circles for strict phagotrophs, orange squares for phago-mixotrophs, green triangles for strict phototrophs and grey triangles for reference organisms lacking both strategies. Test organisms are represented as diamonds. The color gradient within the diamonds illustrates the phagocytosis prediction probability score for each test organism.
